# Diagnostic Value of Pleural Effusion Mononuclear Cells Count and Adenosine Deaminase for Tuberculous Pleurisy Patients in China: A Case-Control Study

**DOI:** 10.3389/fmed.2019.00301

**Published:** 2019-12-17

**Authors:** Xiaoli Lei, Junli Wang, Zhigang Yang, Shengli Zhou, Zhiwei Xu

**Affiliations:** ^1^Department of Respiratory Medicine, Henan Provincial People's Hospital, Henan University People's Hospital, Zhengzhou, China; ^2^Henan University People's Hospital, Henan Provincial People's Hospital, Zhengzhou, China; ^3^Department of Pathology, Henan Provincial People's Hospital, Henan University People's Hospital, Zhengzhou, China; ^4^Department of Clinical-Research Service Center, Henan Provincial People's Hospital, Henan University People's Hospital, Zhengzhou, China

**Keywords:** tuberculous pleurisy, pleural effusion, mononuclear cells count, adenosine deaminase, diagnostic accuracy

## Abstract

**Background:** The diagnostic value of pleural effusion mononuclear cells count for tuberculous pleurisy (TBP) is unclear. We aimed to evaluate the diagnostic value of pleural effusion mononuclear cells count and its combination with adenosine deaminase (ADA) in TBP patients.

**Methods:** We initially analyzed 296 patients with unknown pleural effusion from the Department of Respiratory Medicine at Provincial People's Hospital during January 2014 to February 2018. Ultimately, 100 tuberculous pleurisy (TBP) patients and 105 non-tuberculous pleurisy (non-TBP) patients with pleural effusion were investigated in the current study. Meanwhile, pleural effusion mononuclear cells count and ADA test were performed to evaluate the diagnostic value for TBP. The sensitivity, specificity, positive predictive value (PPV), negative predictive value (NPV), positive likelihood ratio (LR+), negative likelihood ratio (LR–), accuracy and area under the receiver operating characteristic (ROC) curve (AUC) of pleural effusion mononuclear cells count only and its combination with ADA for TBP diagnosis were investigated.

**Results:** (i) The best cut-off value of pleural effusion mononuclear cells count for TBP diagnosis was 969.6 × 10^6^/L, with the sensitivity, specificity and accuracy of 76, 57, and 66%, respectively. (ii) Combination of pleural effusion mononuclear cells count and ADA test suggested diagnostic value for TBP. Specifically, serial test showed the sensitivity, specificity, accuracy of 65, 90, 78%, respectively, whereas parallel test revealed the sensitivity, specificity, accuracy of 92, 45, 68%, respectively. The sensitivity of parallel test (92%) was significantly higher than pleural effusion mononuclear cells count alone (76%) (X^2^ = 23.19, *p* < 0.001). (iii) The area under the ROC of pleural effusion mononuclear cells count and it combined with ADA were 0.66 (95% CI, 0.59–0.72) and 0.83 (95% CI, 0.78–0.89), respectively, with statistically significant difference (Z = 3.46, *p* < 0.001).

**Conclusion:** This retrospective case-control study demonstrated that pleural effusion mononuclear cells count is relatively useful for TBP diagnosis. Furthermore, the pleural effusion mononuclear cells count in combination with ADA can further improve the diagnostic accuracy of TBP.

## Introduction

Tuberculosis (TB) is a serious global public health problem. The World Health Organization estimated that about 10 million people developed TB disease in 2017 globally. China is one of the TB high-risk areas in the world. In 2017, about 889,000 new cases of TB were reported in China, accounting for 9% of new cases worldwide (1). As a common extrapulmonary TB, Tuberculous pleurisy (TBP) accounts for 25% of TB cases in China (2–4). The diagnosis of TBP is difficult (5, 6), with the thoracoscopic pleural pathology as the gold standard method (7). However, thoracoscopic pleural biopsy is an invasive operation with the issues of considerable risks and cost. In addition, yield and complication rate are dependent on the operator's skills (8–10). The operation also has certain limitations in application for elderly patients and hospitals that lack thoracoscopic surgery (10). Further study is needed to investigate less invasive or even non-invasive methods with high accurate diagnosis. Pleural fluid adenosine deaminase (ADA) test is widely used in clinical practice. However, its sensitivity and specificity in the diagnosis of TBP vary greatly (11, 12). Lymphocytes and monocytes in TBP pleural effusions were reported significantly increased. The proportion of lymphocytes and monocytes also had certain clinical significance in the diagnosis of TBP (13, 14). However, lymphocytes and monocytes in pleural effusions are usually not differentiated in clinical practice. Currently, automated blood analyzers are widely used to count mononuclear cells, which include lymphocytes and monocytes (15). Therefore, we hypothesized that, combined with ADA test, pleural effusion mononuclear cells count may contribute to the diagnosis of TBP. This retrospective case-control study aimed to evaluate the diagnostic value of pleural effusion mononuclear cells count and its combination with ADA in TBP patients. We hope to provide a new method for the diagnosis of TBP that is accurate, simple and less invasive.

## Materials and Methods

### Study Design and Subjects

**Figure 1:**
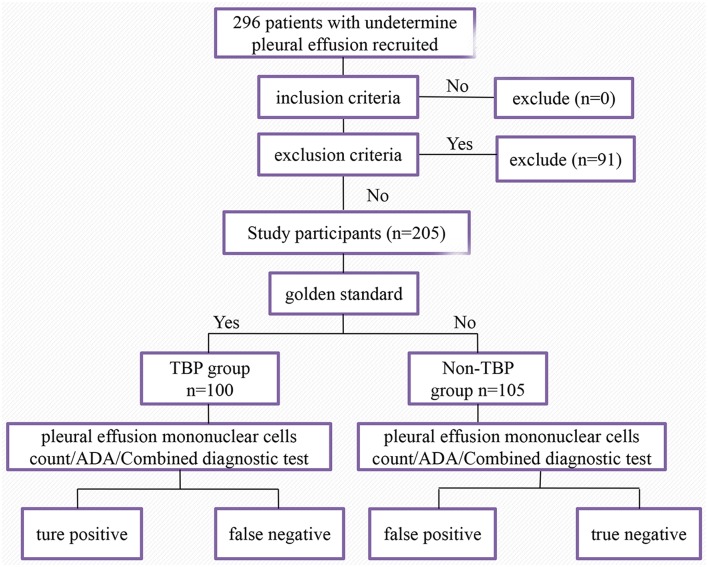
Flowchart of the study population. A total of 296 patients with undetermined pleural effusion were recruited initially. Two hundred and five patients were included in the final analysis.

This retrospective case-control study was conducted using pleural effusion samples from 296 patients between January 1, 2014 and February 28, 2018 from Henan Provincial People's Hospital in China. Samples that meet all the following criteria were included: (i) Pleural effusion was indicated by either chest X-ray, chest CT or ultrasound; (ii) Etiology of pleural effusion was undetermined; (iii) Histopathological examination of pleural tissue was performed after obtained through thoracoscopy; (iv) Results of pleural effusion mononuclear cells count and ADA test were obtained; (v) Complete clinical data of the study subjects were acquired. In this study, patients were divided into TBP group and non-tuberculous pleurisy (non-TBP) group by the gold standard of the pathological results of thoracoscopic pleural biopsy (Figure 1).

TBP was diagnosed based on any of the criteria as follows (16–18): (i) Pleural biopsy showed granulomatous inflammation with or without staining of mycobacteria; (ii) *Mycobacterium Tuberculosis* (*Mtb*) was positive in pleural by using acid-fast bacilli staining, culture or PCR. Diagnostic criteria for non-tuberculous pleural effusion included: (i) There was no microbiological or histological findings of TB; (ii) Tumor cases were confirmed by pathology or cytology results; (iii) Other cases were diagnosed based on patient symptoms, signs, etiology, imaging findings, and clinical efficacy.

In addition, following medical record information was collected: (i) Basic information of patients, including gender, age, and basic diseases; (ii) Results of thoracoscopic pleural histopathology; (iii) Mononuclear cells count in pleural effusion; (iv) ADA test in Pleural effusion; (v) *Mtb* culture results of pleural tissues or pleural effusin. Basic information, Mononuclear cells count and ADA of patients can be seen in Supplementary Table 1.

### Test Methods

Thoracoscopy examination of all the enrolled subjects was performed by using Olympus LTF-240 (Olympus Corporation, Japan) in the bronchoscopy room at Henan Provincial People's Hospital. Briefly, the puncture point was determined by chest imaging or ultrasound findings. Conventional disinfection was conducted followed by local anesthesia with 10 ml 2% lidocaine. The standard thoracoscopic procedure was then performed. Finally samples for biopsy at multiple sites in the parietal pleural lesion was taken for further examination. Pleura specimens were fixed with 4% formaldehyde, and followed by HE staining and acid-fast staining. PCR was also applied to detect *Mtb* by PCR instrument ABI 7500 (ABI, USA).

Pleural effusion mononuclear cells count, pleural effusion ADA test, and pleural effusion TB culture test were conducted in accordance with standard operating procedures strictly. Pleural effusion ADA was determined using Siemens ADVIA2400 biochemical analyzer (Siemens, Germany). The pleural effusion mononuclear cells count was measured by Sysmex xn-9000 automatic blood analyzer (Sysmex Corporation, Japan). The pleural effusion *Mtb* culture was performed using BACTECMGIT 960 rapid culture instrument (BD Corporation, USA).

Sample volume was calculated according to the formula of the sample size for diagnostic study *n* = [Ua2P(1-P)]/δ2 (19). 1.96 is the Ua value for a 95% confidence level. P represents sensitivity when used to calculate patients group and specificity when used to calculate the control group. δ is the allowable error, and usually is set at 0.05. Previous studies showed that the sensitivity and specificity of pleural effusion ADA in the diagnosis of tuberculous pleurisy are 93.6 and 90.9%, respectively (20). Our current study eventually included 205 patients, including 100 TBP patients.

### Blind Method

The pleural effusion mononuclear cells count and pleural effusion ADA test were operated by laboratory technicians independently. Pleural pathological results were judged independently by two pathologists. The third pathologist would be involved when the opinions were inconsistent. The results of subjects' pleural pathology were unknown to laboratory technicians. The pleural effusion mononuclear cell count and ADA test results were unknown to pathologists either.

### Pleural Effusion Mononuclear Cells Count and ADA Test Combination

The receiver operating characteristic (ROC) curve of pleural effusion mononuclear cells count and ADA level for diagnosis of TBP was plotted. When the index was the maximum, the corresponding was the optimal critical value. If the count of pleural effusion mononuclear cells and ADA level were greater than their respective optimal critical values, the diagnosis result was determined to be positive. The combination test includes parallel test and serial test. Parallel test means that two screening tests performed at the same time and the results are subsequently combined. In our current study, parallel test refers to pleural effusion mononuclear cell count and pleural effusion ADA were test simultaneously. The diagnosis is positive as long as one of the results is positive. Serial test means that second screening test is performed only if the result of the first screening test is positive. In our current study, it refers to the pleural effusion mononuclear cell count and pleural effusion ADA were tested serially in order to conduct diagnosis. The combined diagnosis is positive only when the results of both tests are positive.

### Statistical Analysis

Statistical analysis was performed using SPSS version 22.0 (SPSS Inc., Chicago, IL, USA). Continuous variables were defined means ± standard deviation; categorical variables were given as percentage. The independent sample t test or the Mann-Whitney U test was used for the continuous variables and the chi-square test for categorical variables. Diagnostic performance was evaluated using sensitivity, specificity, positive likelihood ratio (LR+), negative likelihood ratio (LR-), positive predictive value (PPV), and negative predictive value (NPV). ROC curves were plotted to assess the diagnostic performance of pleural effusion mononuclear cells and ADA followed by areas under the ROC curve (AUCs) calculation. In addition, optimal cut-off values were obtained by ROC analysis, and ROC analysis based on the multivariate logistic regression model was conducted to assess the diagnostic value of the combined assays. Significance was inferred for *p* < 0.05.

## Results

### Study Patients

A total of 296 patients with undetermined pleural effusion were enrolled initially. Ninety one patients were excluded for lack of results either thoracoscopic pleural biopsy, pleural effusion mononuclear cells count, or ADA. Finally a total of 100 TBP patients (70 males and 30 females) and 105 non-TBP patients (75 males and 30 females) were investigated in this study. Clinical characteristics of 205 patients with pleural effusion can be seen in Table 1. TBP group consisted of one tuberculous pyopneumothorax patient, one tuberculous empyema patient and six TBP patients with pulmonary TB. Details of diseases classification for 105 non-TBP patients with pleural effusion can be seen in Table 2.

**Table 1:** Clinical characteristics of 205 patients with pleural effusion.

**Characteristics**	**TBP group**	**Non-TBP group**	***p*-value**
	**(*n* = 100)**	**(*n* = 105)**	
Age, y (mean ± SD)	45.9 ± 18.6	61.0 ± 14.3	<0.001
**Gender (*****n*****, %)**			
Male	70 (70)	75 (71.4)	0.822
Female	30 (30)	30 (28.6)	
**Underlying condition or illness (*****n*****, %)**			
Alcoholism	26 (26)	34 (32.4)	0.316
Tobacco	37 (37)	53 (50.1)	0.052
Diabetes	8 (8)	19 (18.1)	0.033
Hypertension	22 (22)	24 (22.9)	0.883
Arrhythmia	1 (1)	8 (7.6)	0.049
Coronary heart disease	2 (2)	10 (9.5)	0.022
Chronic gastritis	0 (0)	8 (7.6)	0.014
Brain infarction	5 (5)	10 (9.5)	0.214
COPD	0 (0)	2 (1.9)	0.498
Bronchial asthma	1 (1)	2 (1.9)	1.000
Rheumatologic disease	1 (1)	5 (4.8)	0.237
Hyperthyroidism	1 (1)	1 (1)	1.000
Solid tumor	3 (3)	1 (1)	0.579
Chronic viral hepatitis B	2 (2)	4 (3.8)	0.723
Previous TB infection history	0 (0)	4 (3.8)	0.143
Prior TB treatment	11 (11)	12 (11.4)	0.923
Prior glucocorticoid use	2 (2)	0 (0)	0.237

*TBP, tuberculous pleurisy; Non-TBP, non-tuberculous pleurisy*.

**Table 2:** Non-tuberculous pleurisy patients with pleural effusion diseases classification.

**Classification of diseases**	**Number**	**Proportion (%)**
Empyema	11	10.48
Pneumonia effusion	23	21.90
Pulmonary embolism	1	0.95
Pulmonary contusion	1	0.95
Thoracic cyst	1	0.95
Sepsis	1	0.95
Liver cirrhosis	1	0.95
Microscopic polyangiitis	1	0.95
Nephrotic syndrome	1	0.95
Acute glomerulonephritis	1	0.95
Constrictive pericarditis	2	1.90
Heart failure	4	3.81
Hypoalbuminemia	1	0.95
Malignant pleural effusion	56	53.33

### Diagnostic Value of Pleural Effusion Mononuclear Cells Count

When the largest index of the pleural effusion mononuclear cells count for TBP diagnosis was 0.33, the best cut-off of TBP was 969.6 × 10^6^/L. The sensitivity, specificity, positive predictive value, negative predictive value, accuracy, positive likelihood ratio, negative likelihood ratio, and were 76, 57, 63, 71, 66%, 1.77, and 0.42, respectively (Table 3).

**Table 3:** Diagnostic performance of pleural effusion mononuclear cells count, ADA and combination diagnostic test (*n* = 205).

	**Sensitivity (%)**	**Specificity (%)**	**PPV**	**NPV**	**LR+**	**LR–**	**Accuracy (%)**
Mononuclear cells count	76^*^	57^#^	0.63	0.71	1.77	0.42	66
ADA (>27 U/L)	81	78	0.78	0.81	3.70	0.24	80
Serial test	65	90^#^	0.87	0.73	6.83	0.39	78
Parallel test	92^*^	45	0.61	0.85	1.67	0.18	68

PPV, positive predictive value; NPV, negative predictive value; LR+, likelihood ratio for positive test; LR-, likelihood ratio for negative value;

# X^2^ = 12.27, p = 0.000, comparison of the specificity among pleural effusion mononuclear cells and Serial test;

* *X^2^ = 23.19, p = 0.000, comparison of the sensitivity among pleural effusion mononuclear cells and Parallel test*.

### Diagnostic Value of ADA

When the largest index of the ADA for TBP diagnosis was 0.59, the best cut-off of ADA was 27 U/L. The sensitivity, specificity, positive predictive value, negative predictive value, accuracy, positive likelihood ratio and negative likelihood ratio were 81, 78, 78, 81, 80%, 3.70, and 0.24, respectively (Table 3).

### Diagnostic Value of the Combination of Pleural Effusion Mononuclear Cells Count and ADA

The sensitivity, specificity, positive predictive value, negative predictive value, accuracy, positive likelihood ratio, and negative likelihood ratio of the serial test were 65, 90, 87, 73, 78%, 6.83, 0.39, respectively; Above evaluation indexes of parallel test were 92, 45, 61, 85, 68%, 1.67, 0.18, respectively (Table 3).

The specificity of serial test that pleural effusion mononuclear cells count combined with ADA (90%) for TBP diagnosis was significantly higher than that of pleural effusion mononuclear cells count alone (57%) (X^2^ = 12.27, *p* = 0.000), the sensitivity (65%) was lesser than that of pleural effusion mononuclear cells count (76%) (X^2^ = 58.65, *p* = 0.000); The sensitivity of parallel test that pleural effusion mononuclear cells count combined with ADA (92%) for the diagnosis of TBP was significantly higher than that of pleural effusion mononuclear cells count (76%) (X^2^ = 23.19, *p* = 0.000), the specificity (45%) was lower than that of pleural effusion mononuclear cells count (57%) (X^2^ = 63.82, *p* = 0.000) (Table 3).

**Figure 2:**
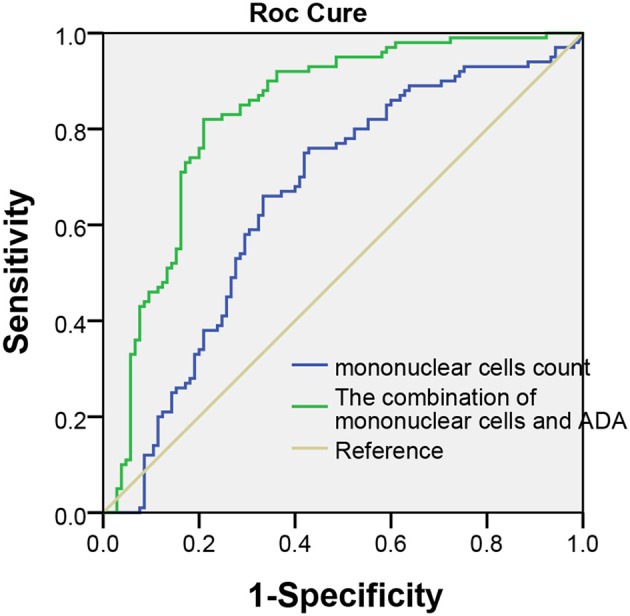
Receiver operating characteristic curve in pleural effusion mononuclear cells and pleural effusion mononuclear cells count combination with ADA for TBP diagnosis. The area under the ROC of pleural effusion mononuclear cells and combined test was 0.66 (95% CI, 0.59–0.72), 0.83 (95% CI, 0.78–0.89) (Z = 3.46, *p* < 0.05).

The area under the ROC of pleural effusion mononuclear cells count and pleural effusion mononuclear cells count combined with ADA for TBP was 0.66 (95% CI, 0.59–0.72), 0.83 (95% CI, 0.78–0.89), respectively. It was statistically significant difference in area under ROC curve between the pleural effusion mononuclear cells count and the combination test for TBP diagnosis (Z = 3.46, *p* < 0.001) (Figure 2).

## Discussion

Cellular immunity is the most important immune protection mechanism for Mycobacteria infection. Macrophages in the alveoli secrete a large number of interleukin-1 (IL-1), interleukin-6 (IL-6), tumor necrosis factor-α (TNF-α), and other cytokines, so that lymphocytes and monocytes are accumulated in mycobacterial invasion sites and followed by granulomas formation. TBP could infect the pleura through various routes, causing exudation, hyperplasia, and necrotic inflammation which mainly consisted of lymphocyte and monocyte infiltration. Unlike TB, patients with tuberculous pleural effusion usually have an acute febrile illness with nonproductive cough and pleuritic chest pain, night sweats, chills, weakness, dyspnea, and weight loss can also occur (4, 21). If it is not diagnosed and treated in time, TBP often leads to serious complications such as pleural thickening, calcification, empyema, bronchopleural fistula, etc. (22, 23). However, due to the lack of specificity of the clinical features of this disease, it is difficult to distinguish TBP from malignant pleural effusion and pneumonia-like pleural effusion, making the diagnosis difficult.

Transthoracic pleural biopsy is commonly used in recent years. Pleural pathology showing granulomatous inflammation with acid-fast staining or positive culture of *Mtb* is the gold standard for the diagnosis of TBP (16–18). However, the thoracoscopic pleural biopsy is invasive and expensive operation with certain surgical trauma and postoperative complications. Some elderly patients cannot tolerate the procedure. In addition, a large number of patients in China with TBP are admitted to primary or secondary hospitals lack of thoracoscopy equipment, which made TBP difficult to be diagnosed in a timely manner for the patients in those hospitals. More accurate, simple, and less invasive diagnostic methods are urgently needed. TBP patients often have pleural effusion during the active phase. Therefore, the diagnosis of TBP through thoracentesis with the assistance of cell types measurement, various enzymes and inflammatory factors in pleural effusion has become a hot topic of research.

Lymphocytes and monocytes in pleural effusion increase significantly after Mtb infection (13, 14). The proportion of lymphocytes and the ratio of monocytes have certain diagnostic value for the diagnosis of tuberculous pleurisy. The literature reported that when the pleural effusion lymphocyte is >64%, the sensitivity and specificity of diagnosis of TBP could reach to 89.1 and 76.4% (24). Another report revealed that the sensitivity and specificity of the proportion of lymphocytes (LP ≥ 50%) in pleural effusion combined with ADA greater or equal to 40 U/L for TBP were 86.3 and 98.3%, respectively (13). In addition, the literature reported the proportion of pleural fluid monocytes cells (%) was 84.5 combined with the pleural effusion ADA value was 28.7 U/L (14), the sensitivity and specificity for TBP were 57.5 and 98%, respectively. The above results suggest that the proportion of lymphocytes and the proportion of monocytes are helpful in the diagnosis of TBP. However, lymphocytes and monocytes in clinical pleural effusions are usually not separately measured. The automatic blood analyzer is widely used to detect the count of mononuclear cells (the main components are lymphocytes, monocytes and a small number of mononuclear cells from other sources), which is simple, rapid and accurate (25, 26). Therefore it has been used as a routine examination item in clinical laboratories. We hypothesized that pleural effusion mononuclear cells count might contribute to the TBP diagnosis. Our study showed that the best cut-off of pleural effusion mononuclear cells count was 969.6 × 10^6^/L. Meanwhile, the sensitivity, specificity, accuracy, area under the ROC of pleural effusion mononuclear cells count for TBP were 76, 57, 66%, 0.66 (95%CI, 0.59–0.72), respectively. Our results suggested the value of pleural effusion mononuclear cells count in the diagnosis of TBP for the first time. To the best of our knowledge, similar studies have not been published.

The pleural effusion ADA is of great value in the diagnosis of TBP (11). In this study, the sensitivity, specificity and accuracy for diagnosis of TBP were 81, 78, and 80%, respectively. The best cut-off value of ADA for TBP was 27 U/L, which is similar to 26.5 U/L (27). Garcia-Zamalloa's study suggested the best cut-off value of ADA for the diagnosis of TBP was 40 U/L (13). The reasons of the difference are not clear yet. Study population, different prevalence rates of TBP, as well as sample sizes might contribute to the difference, further investigation is needed in the future.

This study further explored combination of pleural effusion mononuclear cells count and ADA test in order to improve the accuracy of TBP diagnosis. When combination was used, the area under ROC curve of combination was 0.83 (95% CI, 0.78–0.89), which was significantly higher than that of 0.66 (95% CI, 0.59–0.72) when pleural effusion mononuclear cells count was used only (*p* < 0.05). The difference suggested that combination of pleural effusion mononuclear cells count and ADA test were better than pleural effusion mononuclear cells count was used alone for TBP diagnosis. Our studies also showed that the sensitivity and specificity of combination test was significantly higher than that of sensitivity and specificity when pleural effusion mononuclear cells count was used only for TBP diagnosis (*p* < 0.05). Parallel test has higher sensitivity but lower specificity; as comparison, serial test improves specificity at the cost of lower sensitivity, which was consistent with our results. The likelihood ratio increased from 1.77 to 6.83 when the serial test was used to analyze the results, which indicated that compared with single nuclear cells count, serial test can significantly improve diagnostic value of TBP. Meanwhile, the negative likelihood ratio decreased from 0.42 to 0.18 when the parallel test was used to analyze the results, which indicated that parallel test is more appropriate used to exclude the diagnosis of TBP. The thoracic pleural thickening, adhesion, and even pleural calcification of the affected side lead to thoracic collapse could happen, causing irreversible restrictive ventilation dysfunction if TBP is misdiagnosed. Therefore, it is more important to reduce the rate of misdiagnosis and improve the sensitivity of the diagnostic test. As a result, we recommended parallel test for pleural effusion mononuclear cell count combined with ADA.

In this study, the two study groups were different in age and chronic diseases, such as diabetes, arrhythmia, coronary heart disease, chronic gastritis. One study indicated that pleural tuberculosis was most commonly seen in adolescents and young adults (28), which was similar with this study. Another study found out that, compared with tuberculosis patients who had diabetes, the incidence of pleurisy is reduced in the tuberculosis patients who do not have diabetes (29). That study suggested that diabetes has a certain impact on the occurrence of TBP. No other chronic diseases (arrhythmia, coronary heart disease, chronic gastritis) have been found to affect TBP. Our findings suggested that future research needs to further study the effects of the above chronic diseases on TBP.

Finally, it is worthwhile to note that the quality of the study might be not as good as the cross-sectional study in the same period due to the characteristic of retrospective study. In addition, the patients in this study were enrolled in grade III hospital in China with relatively severe symptoms. Therefore, the selective bias in the population might occur. Simultaneous cross-sectional studies can be used in the future to further investigate the value of combination test of pleural effusion mononuclear cells count and ADA for the diagnosis of TBP.

In summary, pleural effusion mononuclear cells count is helpful in the diagnosis of TBP in our study. Diagnostic accuracy of TBP was improved when combined with ADA test. Our findings provide a new diagnostic method which is more accurate, simple, and less invasive.

## Data Availability Statement

The raw data supporting the conclusions of this article will be made available by the authors, without undue reservation, to any qualified researcher.

## Ethics Statement

The studies involving human participants were reviewed and approved by Ethics Committee of Henan Provincial People's Hospital. Written informed consent for participation was not required for this study in accordance with the national legislation and the institutional requirements. Written informed consent was not obtained from the individual(s) for the publication of any potentially identifiable images or data included in this article.

## Author Contributions

ZY and ZX contributed conception and design of the study. JW and SZ collected the data and organized the database. JW and XL analyzed the data. XL, ZY, and JW wrote the first draft of the manuscript. All authors contributed to the final version of the manuscript.

### Conflict of Interest

The authors declare that the research was conducted in the absence of any commercial or financial relationships that could be construed as a potential conflict of interest.

## Abbreviations

ADA: adenosine deaminase
TBP: tuberculous pleurisy
Non-TBP: non-tuberculous pleurisy
PPV: positive predictive value
NPV: negative predictive value
LR+: positive likelihood ratio
LR–: negative likelihood ratio
ROC: receiver operating characteristic
AUC: area under the receiver operating characteristic curve.

## References

[1] World Health Organization Global Tuberculosis Report 2018. New York, NY: WHO (2018). p. 34.

[2] Jeon D. Tuberculous pleurisy: an update. Tuberc Respir Dis. (2014). 76:153–9. 10.4046/trd.2014.76.4.153 24851127 PMC4021261

[3] Abrao FC de Abreu IR Miyake DH Busico MA Younes RN. Role of adenosine deaminase and the influence of age on the diagnosis of pleural tuberculosis. Int J Tuberc Lung Dis. (2014) 18:1363–9. 10.5588/ijtld.14.0257 25299872

[4] Zhai K Lu Y Shi HZ. Tuberculous pleural effusion. J Thorac Dis. (2016) 8:E486–94. 10.21037/jtd.2016.05.87 27499981 PMC4958858

[5] Tadele A Beyene D Hussein J Gemechu T Birhanu A Mustafa T. Immunocytochemical detection of *Mycobacterium tuberculosis* complex specific antigen, MPT64, improves diagnosis of tuberculous lymphadenitis and tuberculous pleuritis. BMC Infect Dis. (2014) 14:585. 10.1186/s12879-014-0585-1 25421972 PMC4262190

[6] Kim MC Kim SM Lee SO Choi SH Kim YS Woo JH. A diagnostic algorithm for tuberculous pleurisy using the ELISPOT assay on peripheral blood and pleural effusion. Infect Dis. (2016) 48:688–94. 10.1080/23744235.2016.1183816 27187759

[7] Liu F Gao M Zhang X Du F Jia H Yang X. Interferon-gamma release assay performance of pleural fluid and peripheral blood in pleural tuberculosis. PLoS ONE. (2013) 8:e83857. 10.1371/journal.pone.0083857 24386296 PMC3873962

[8] Trajman A Pai M Dheda K van Zyl Smit R Zwerling AA. Novel tests for diagnosing tuberculous pleural effusion: what works and what does not? Eur Respir J. (2008) 31:1098–106. 10.1183/09031936.00147507 18448504

[9] Rahman NM Ali NJ Brown G Chapman SJ Davies RJ Downer NJ. Local anaesthetic thoracoscopy: British Thoracic Society Pleural Disease Guideline 2010. Thorax. (2010) 65(Suppl 2):ii54–60. 10.1136/thx.2010.137018 20696694

[10] Dixon G de Fonseka D Maskell N. Pleural controversies: image guided biopsy vs. thoracoscopy for undiagnosed pleural effusions? J Thorac Dis. (2015) 7:1041–51. 10.3978/j.issn.2072-1439.2015.01.36 26150917 PMC4466430

[11] Gui X Xiao H. Diagnosis of tuberculosis pleurisy with adenosine deaminase (ADA): a systematic review and meta-analysis. Int J Clin Exp Med. (2014) 7:3126–35. 25419343 PMC4238476

[12] Aggarwal AN Agarwal R Sehgal IS Dhooria S. Adenosine deaminase for diagnosis of tuberculous pleural effusion: a systematic review and meta-analysis. PLoS ONE. (2019) 14:e0213728. 10.1371/journal.pone.0213728 30913213 PMC6435228

[13] Garcia-Zamalloa A Taboada-Gomez J. Diagnostic accuracy of adenosine deaminase and lymphocyte proportion in pleural fluid for tuberculous pleurisy in different prevalence scenarios. PLoS ONE. (2012) 7:e38729. 10.1371/journal.pone.0038729 22723878 PMC3377686

[14] Zhang X Tong C Diagnostic value of hydrothorax mononuclear cell and ADA in tuberculous pleuritis. Med J Chin People's Health. (2014) 12:6–8. 10.3969/j.issn.1672-0369.2014.12.003

[15] Zhu L Chen X Common Experimental Methods of Immunology. Beijing: People's Military Medical Press (2000). p. 153–5.

[16] Gopi A Madhavan SM Sharma SK Sahn SA. Diagnosis and treatment of tuberculous pleural effusion in 2006. Chest. (2007) 131:880–9. 10.1378/chest.06-2063 17356108

[17] Losi M Bossink A Codecasa L Jafari C Ernst M Thijsen S. Use of a T-cell interferon-gamma release assay for the diagnosis of tuberculous pleurisy. Eur Respir J. (2007) 30:1173–9. 10.1183/09031936.00067307 17715165

[18] Aggarwal AN Agarwal R Gupta D Dhooria S Behera D. Interferon gamma release assays for diagnosis of pleural tuberculosis: a systematic review and meta-analysis. J Clin Microbiol. (2015) 53:2451–9. 10.1128/JCM.00823-15 25994163 PMC4508404

[19] Liu X Wang S editor. Clinical Epidemiology and Evidence Based Medicine, 4th ed. Beijing: People's Medical Publishing House (2016). p. 132.

[20] Wang H Yue J Yang J Gao R Liu J. Clinical diagnostic utility of adenosine deaminase, interferon-γ, interferon-γ-induced protein of 10 kDa, and dipeptidyl peptidase 4 levels in tuberculous pleural effusions. Heart Lung. (2012) 41:70–5. 10.1016/j.hrtlng.2011.04.049 21917315

[21] Bhuniya S Arunabha DC Choudhury S Saha I Roy TS Saha M. Role of therapeutic thoracentesis in tuberculous pleural effusion. Ann Thorac Med. (2012) 7:215–19. 10.4103/1817-1737.102176 23189098 PMC3506101

[22] Candela A Andujar J Hernández L Martín C Barroso E Arriero JM. Functional sequelae of tuberculous pleurisy in patients correctly treated. Chest. (2003) 123:1996–2000. 10.1378/chest.123.6.1996 12796180

[23] Kumar A Asaf BB Lingaraju VC Yendamuri S Pulle MV Sood J. Thoracoscopic decortication of stage III tuberculous empyema is effective and safe in selected cases. Ann Thorac Surg. (2007) 104:1688–94. 10.1016/j.athoracsur.2017.06.038 28964422

[24] Sahn SA Huggins JT San José ME Álvarez-Dobaño JM Valdés L. Can tuberculous pleural effusions be diagnosed by pleural fluid analysis alone? Int J Tuberc Lung Dis. (2013) 17:787–93. 10.5588/ijtld.12.0892 23676163

[25] Fleming C Brouwer R Lindemans J de Jonge R. Validation of the body fluid module on the new Sysmex XN-1000 for counting blood cells in cerebrospinal fluid and other body fluids. Clin Chem Lab Med. (2012) 50:1791–8. 10.1515/cclm-2011-0927 23089709

[26] Buoro S Mecca T Azzarà G Seghezzi M Dominoni P Crippa A. Cell population data and reflex testing rules of cell analysis in pleural and ascitic fluids using body fluid mode on Sysmex XN-9000. Clin Chim Acta. (2016) 452:92–8. 10.1016/j.cca.2015.11.005 26554518

[27] Xu HY Zhang DQ Ye JR Su SS Xie YP Chen CS. Diagnostic performance of T-SPOT.TB on peripheral blood in combination with adenosine deaminase on pleural fluid for the diagnosis of tuberculous pleurisy within different age group. Zhonghua Yi Xue Za Zhi. (2017) 97:1862–6. 10.3760/cma.j.issn.0376-2491.2017.24.004 28648009

[28] Chakrabarti B Davies PD. Pleural tuberculosis. Monaldi Arch Chest Dis. (2006) 65:26–33. 10.4081/monaldi.2006.582 16700190

[29] Mamaev IA Musaeva AM Abusuev SA Mamaeva KhI Untilov GV The epidemiological features of concomitance of diabetes mellitus and pulmonary tuberculosis. Probl Tuberk Bolezn Legk. (2008) 5:23–5. 18711815

